# Editorial: Natural products as sources of innovative approaches in the prevention and treatment of neuronal injuries induced by neurodegenerative diseases and environmental exposures to neurotoxic agents

**DOI:** 10.3389/fncel.2023.1206576

**Published:** 2023-06-06

**Authors:** Fernando Allan de Farias Rocha

**Affiliations:** Laboratory of Neurophysiology Eduardo Oswaldo Cruz, Institute of Biological Science, Federal University of Pará, Belém, Pará, Brazil

**Keywords:** neurodegenerative diseases, natural products, antioxidant, anti-inflammatory, innovative approaches

Currently, natural products promote a promising way to prevent neural damage from neurodegenerative diseases. Although modern medicine provides drugs that show some effectiveness in the treatment of neural damage, natural products provide an alternative with low side effects that, when incorporated into the diet, can promote neuroprotection of the central nervous system.

In this scenario, the prevention of damage caused by diseases in the nervous system is essential for patients with chronic diseases involving tissue degeneration. Therefore, the use of natural products with recognized antioxidant action can be a good preventive approach.

Thus, the objective of this Research Topic is to gather studies that show the effectiveness of natural products in preventing damage caused by neurodegenerative diseases and/or toxic agents in order to seek innovative solutions for the prevention of neuronal injuries.

The scope of the Research Topic consists of articles on the use of natural products in the prevention and/or treatment of neuronal damage caused by neurodegenerative diseases and neurotoxic agents. We will now present the accepted articles and the contributions of each of the works.

The authors investigated the antioxidant and anti-inflammatory effects of calcitriol, the most active form of vitamin D3; the study aimed to explore the protective and restorative effects of calcitriol in rotenone-induced Parkinson's disease (Magdy et al.). The effect of calcitriol on motor impairment and balance in rats with Parkinson's disease was tested. Additionally, a morphological investigation of the cytoarchitecture and histopathological features in the midbrain of rats with Parkinson's disease was performed. The research results were very promising as they showed that behavioral impairments and depletion of dopaminergic neurons in patients induced in the Parkinson's disease model were ameliorated by the administration of calcitriol. Furthermore, the results of the study may help to complement and optimize an efficient therapeutic strategy for the treatment of Parkinson's disease.

The endocannabinoid system was the subject of a review published for this Research Topic (Paes-Colli et al.). The authors enriched the discussion and clarified the benefits of using cannabis in diseases such as Parkinson's, Alzheimer's disease and Multiple Sclerosis. In the review, we highlight that in pre-clinical studies, conducted *in vitro* and *in vivo*, it has already been extensively demonstrated that phytocannabinoids and Cannabis-based medicine can reverse altered cellular, molecular, and behavioral aspects in models of Parkinson's, Alzheimer's, and Multiple Sclerosis. In Alzheimer's disease, studies have reported a reduction in astrocytic reactivity, neuroinflammation, memory loss, and cognitive scores, while in Parkinson's disease, a reduction in cell death of dopaminergic neurons and neuroinflammation has been observed to be associated with the recovery of motor and cognitive capacity in animals. Thus, the aim of this review is to focus on the importance of the endocannabinoid system in these diseases and how phytocannabinoids can be used in their treatment in clinical practice.

Açaí (*Euterpe oleracea Mart*.) was the subject of an article that addressed electrophysiological and behavioral techniques to evaluate the effect of açaí in epilepsy models (Muto et al.). The data presented showed that açaí produced an increase in seizure latency and the non-appearance of behavioral changes, such as generalized clonic seizures with transient loss of postural reflex and tonic-clonic seizures with loss of postural reflex. These results indicate that the compounds present in açaí are effective in increasing the seizure threshold for the epilepsy model, which was also observed in electroencephalographic recordings.

Finally, in another study involving a model of epilepsy, the authors brought an unprecedented approach, evaluating the effectiveness of treatment with Curcuma longa, a herbal medicine, in association with diazepam, a drug often used in the treatment of epilepsy. In the study, it was shown that pre-treatment with Curcuma longa associated with diazepam attenuated the electroencephalographic tracing and seizure control with a reduction in the frequency and amplitude of peak waves. In behavioral observation, the animals did not develop tonic-clonic seizures, protecting the hippocampus cells. With these results it was shown that Curcuma longa in association with diazepam can be a therapeutic option in the future for the treatment of epilepsy (Nascimento et al.).

In view of the works presented above, we were able to show with this Research Topic that there are natural products that might be used in the near future in the efficient treatment of various neurodegenerative diseases.

## Author contributions

The author confirms being the sole contributor of this work and has approved it for publication.

